# Pharmacogenetic clinical randomised phase II trial to evaluate the efficacy and safety of FOLFIRI with high-dose irinotecan (HD-FOLFIRI) in metastatic colorectal cancer patients according to their *UGT1A 1* genotype

**DOI:** 10.1038/s41416-018-0348-7

**Published:** 2018-12-26

**Authors:** David Páez, María Tobeña, Julen Fernández-Plana, Ana Sebio, Anna C. Virgili, Lluís Cirera, Agustí Barnadas, Pau Riera, Ivana Sullivan, Juliana Salazar

**Affiliations:** 1 Medical Oncology Department, Hospital de la Santa Creu i Sant Pau, Barcelona, Spain; 2 CIBERER U-705, Barcelona, Spain; 3 Medical Oncology Department, Hospital Universitario Mutua Terrassa, Terrassa, Spain; 4 Universitat Autònoma de Barcelona (UAB), Barcelona, Spain; 5 Genetics Department, Hospital de la Santa Creu i Sant Pau, Barcelona, Spain; 6 Universitat de Barcelona (UB), Barcelona, Spain

**Keywords:** Chemotherapy, Colorectal cancer

## Abstract

**Background:**

Patients harbouring the UGT1A1*28/*28 genotype are at risk of severe toxicity with the standard irinotecan dose. However, this dose is considerably lower than the dose that can be tolerated by UGT1A1*1/*1 and *1/*28 patients. This randomised phase II trial evaluated the efficacy and safety of the FOLFIRI regimen with high-dose irinotecan (HD-FOLFIRI) in metastatic colorectal cancer patients.

**Methods:**

Eighty-two patients with the UGT1A1*1/*1 or the *1/*28 genotype were randomised to receive HD-FOLFIRI versus FOLFIRI. Patients with the UGT1A1*28/*28 genotype were excluded. In the experimental group, the irinotecan dose was 300 mg/m^2^ for UGT1A1*1/*1 and 260 mg/m^2^ for *1/*28 patients. In the control group, the dose was 180 mg/m^2^. We analysed the overall response rate (ORR), toxicity, and survival.

**Results:**

The ORR was significantly higher in the HD-FOLFIRI group (67.5 versus 43.6%; *p* = 0.001 OR: 1.73 [95% CI:1.03–2.93]). Neutropenia (17.7%), diarrhoea (5.1%), and asthenia (5.1%) were the most common grade 3–4 toxicity. No differences were observed in severe toxicity (22.5% versus 20.5%), dose reduction (22.5% versus 28.2%), or prophylactic G-CSF (17.5% versus 12.8%). No difference in survival was found.

**Conclusions:**

Patients with the UGT1A1*1/*1 and *1/*28 genotypes can receive high doses of irinotecan to achieve a more favourable ORR without significant adverse events.

## Introduction

Colorectal cancer (CRC) is the second most frequent neoplasm in industrialised countries. Worldwide, it is the fourth deadliest type of cancer. At initial diagnosis, around 30% of patients have inoperable or metastatic disease, and >50% of patients will receive chemotherapy at some stage of the disease.^1,2^ Current cytotoxic agents are fluoropyrimidine-based regimens in combination with oxaliplatin or irinotecan.^3^

Irinotecan (CPT11) inhibits topoisomerase I, an enzyme needed to separate the DNA double helix during replication and transcription.^4^ This inhibition leads to cell death and is the basis of its antineoplastic effect. Irinotecan is converted to an active metabolite, 7-ethyl-10-hydroxycamptothecin (SN-38) by a carboxylesterase, and finally metabolised through the action of uridine diphosphate glucuronosyltransferase (UGT) enzymes. The predominant enzyme is UGT1A1, the enzyme that conjugates bilirubin.^5^ In the promoter region of the *UGT1A1* gene, an extra TA dinucleotide characterises the genotype associated with Gilbert’s syndrome (chronic non-conjugated hyperbilirubinemia due to reduced UGT1A1 activity). The presence of this polymorphism (TA7) results in the UGT1A1*28 variant allele instead of the dominant allele of 6 TA repeats (UGT1A1*1). Patients who are homozygous for this variant (UGT1A1*28/*28) have less enzymatic activity and are predisposed to develop myelosuppression and severe diarrhoea when treated with irinotecan.^6–12^ Premature drug suspension and dose reduction as well as administration delays due to toxicity can decrease antitumour activity. Therefore, serious toxicity rates that decrease survival can be anticipated through genetic analysis of patients prior to treatment, thereby optimising and personalising irinotecan dosing. The United States of America Food and Drug Administration recognises the importance of *UGT1A1* pharmacogenetics in predicting toxicity to irinotecan and describes the association between reduced enzymatic activity of the UGT1A1*28 allele and neutropenia after drug administration in their summary of product characteristics. The recommendation is to reduce the dose of irinotecan in homozygous patients.^13^ Despite the good will of this recommendation, however, the exact dose reduction needed to limit drug toxicity is not specified for *28/*28 patients.

In a previous study (EC07/90232), we classified patients with metastatic CRC (mCRC) treated with first-line chemotherapy (FOLFIRI regimen) according to their *UGT1A1* genotype. The goal was to predict, diminish, and/or avoid toxicity and improve the therapeutic effect of chemotherapy. This optimisation was based on administering different doses depending on the genotype of the *UGT1A1* gene. The results showed that the recommended dose of irinotecan within the FOLFIRI regimen (180 mg/m^2^) was considerably lower than the dose tolerated by UGT1A1*1/*1 and *1/*28 patients.^14^ These findings validated the results reported by an Italian group who performed a clinical phase I irinotecan dose-escalation trial according to the *UGT1A1* genotype in mCRC patients treated with FOLFIRI.^15^ Based on these two studies, it has been proposed to individualise irinotecan dosing according to the *UGT1A1* status so as to optimise treatment efficacy and tolerability. However, whether genotype-driven dosing will lead to differences in outcome has yet to be tested prospectively. For these reasons, the main objectives of the present randomised phase II trial were to evaluate the efficacy and safety of the FOLFIRI regimen with high-dose irinotecan (HD-FOLFIRI) in mCRC patients with a favourable *UGT1A1* genotype (homozygous wild type *1/*1 and heterozygous *1/*28), while excluding patients genetically at risk for toxicity (*28/*28).

## Methods

### Study design

This randomised, multicentre, open-label, non-blinded phase II study was conducted in three hospitals in Spain: Hospital de la Santa Creu i Sant Pau, Barcelona; Hospital Universitario Mutua Terrassa, Terrassa; and Hospital de Mataró, Mataró, Barcelona. The protocol was approved by the institutional review board at each participating centre. All patients signed a written informed consent form before entering the study. The clinicaltrials.gov identifier was NCT01639326.

### Patient eligibility

Patients with histologically confirmed diagnosis of mCRC and measurable disease defined via RECIST (Response Evaluation Criteria in Solid Tumour; version 1.1) were enrolled. Eligibility criteria were: UGT1A1*1/*1 or *1/*28 genotypes, no prior chemotherapy for metastatic disease, age ≥18–< 76 years, The Eastern Cooperative Oncology Group (ECOG) performance status of 0 or 1, absolute neutrophil count ≥1500/μl, platelets ≥100,000/μl, creatinine clearance <1.5× the upper limit of normal (ULN), alanine transaminase and aspartate aminotransferase <2.5× the ULN ( < 5× the ULN in the presence of liver metastases), and total serum bilirubin ≤1.5 mg/dl. Patients with the UGT1A1*28/*28 genotype or carriers of other *UGT1A1* alleles (*6, *36 (TA5), *37 (TA8)) were not eligible.

### Randomisation and drug administration

Between June 2012 and October 2016, patients were randomised 1:1 to receive HD-FOLFIRI (experimental group) versus FOLFIRI (control group) every 2 weeks. Treatment allocation was conducted using block randomisation and stratified by centre. The Statistical Package for Social Sciences, Version 19.0 (SPSS Inc., Chicago, IL, USA) was used. Irinotecan doses for UGT1A1*1/*1 and *1/*28 patients in the experimental group were 300 and 260 mg/m^2^, respectively. These doses were chosen based on our previous phase I trial, in which a dose ≥260 mg/m^2^ was an independent predictor of a better response in mCRC patients with the *1/*1 or *1/*28 *UGT1A1* genotypes.^14^ The standard irinotecan dose of 180 mg/m^2^ was administered in the control group. Irinotecan was administered as an intravenous infusion over 90 min on days 1 and 15, with leucovorin 400 mg/m^2^ administered concomitantly. 5-Fluorouracil was administered as a 400 mg/m^2^ bolus immediately after the irinotecan infusion, followed by 2400 mg/m^2^ over a 46-h continuous infusion on days 1 and 15. A cycle was 28 days. Before irinotecan was started, patients were pretreated with atropine 0.5 mg, dexamethasone 20 mg, and granisetron 1 mg. Diarrhoea was promptly treated at onset with oral intake of loperamide 4 mg, and oral intake of 2 mg for any further episodes. Granulocyte-colony stimulating factors (G-CSF) were allowed in patients who had grade ≥3 neutropenia during previous cycles. Treatment was continued until disease progression, unacceptable toxicity, withdrawal of consent, or investigator’s decision, whichever was earlier. To avoid potential bias regarding the primary objectives of the study, biological agents were not allowed.

### Study objectives

The primary objectives were: (1) to determine the effect of higher doses of irinotecan on the efficacy of FOLFIRI as assessed by overall response rate (ORR); and (2) to evaluate safety according to the NCI Common Terminology Criteria for Adverse Events (version 3.0). Objective tumour response was evaluated by the investigator at each centre using the modified RECIST (version 1.1); no independent review was performed. Tumour response was evaluated every 10 weeks (±2 weeks) according to the standard of care at each centre. Secondary objectives were progression-free survival (PFS) and overall survival (OS). PFS was defined from the time of drug administration to the occurrence of progressive disease or death, whichever occurred first. OS was defined from the time of drug administration to the date of death. Patients not meeting the criteria by the cutoff date were censored at the last contact date.

### Genotyping assays

Genomic DNA was extracted from peripheral leukocytes by the salting-out procedure.^16^ The TA index of the *UGT1A1* promoter was genotyped by fragment sizing. Polymerase chain reaction was performed in a total volume of 25 µl containing template DNA (80 ng/µl), according to Monaghan et al.^17^ The primers used were a forward primer that was modified by the addition of a 50 fluorescent-labelled FAM and an unlabelled reverse primer (UGT-FAM_F; 50-GTCACGTGACACAGTCAAAC-30, UGT_R 50-TTTGCTCCTGCCAGAGGTT-30). The PCR product (TA*1, 98 bp; TA*28, 100 bp), the internal size standard, and Hi-Di formamide (GeneScan 500, Applied Biosystems, Foster City, CA, USA) were mixed. The samples were then run in the ABI Prism 3100 Genetic Analyzer (Applied Biosystems). Fragment sizes were determined by comparison with the internal standard GeneScan 500 using the local Southern algorithm and analysed by the GeneMapper software version 3.5 (Applied Biosystems). Homozygous-dominant and heterozygous- and homozygous-recessive sequenced samples were included on every run as a quality control. Genotypes were assigned based on the number of TA repeats in each allele (i.e., TA*1/TA*1, TA*1/TA*28, and TA*28/TA*28).

*KRAS* and *NRAS* mutations in exons 2, 3, and 4 and *BRAF* V600E mutation were assessed on tumour DNA. Genomic DNA was extracted using the QIAamp DNA FFPE Tissue Kit (Qiagen, Hilden, Germany) as specified by the manufacturer’s instructions. Mutational analysis was performed using standard PCR conditions and primers for exons 2, 3, and 4 of *KRAS* and *NRAS* genes and for exon 15 of *BRAF* gene. The thermal cycling conditions were an initial 12 min at 94°C, followed by 40 cycles of 45 s at 94°C, 45 s at primer annealing temperature of 55°C, 10 min at 72°C, and a final extension of 10 min at 72°C. Each sample underwent capillary electrophoresis on an ABI 3500 Genetic Analyzer (Applied Biosystems).

### Statistical analysis

All data were analysed using the Statistical Package for Social Sciences, Version 19.0, software (SPSS Inc., Chicago, IL, USA). ORR (complete+partial response) was compared between groups using chi-square or Fisher’s exact test for Count Data. Based on the report from Van Cutsem et al., the ORR was reported as 38.7% for the FOLFIRI regimen.^18^ An estimated difference to the order of 30% was assumed between the control and the experimental groups. The target sample size was therefore determined to be 96 patients, distributed between the two groups. The effects of the irinotecan dose on PFS and OS were estimated using the Kaplan–Meier estimator, and differences were tested using the log-rank test. For all comparisons, a two-sided *p* value <0.05 was considered significant, so the level was set at 5% (*α* = 0.05) and the *β* level was set at 20% (*β* = 0.20).

## Results

### Patient population

**Fig. 1:**
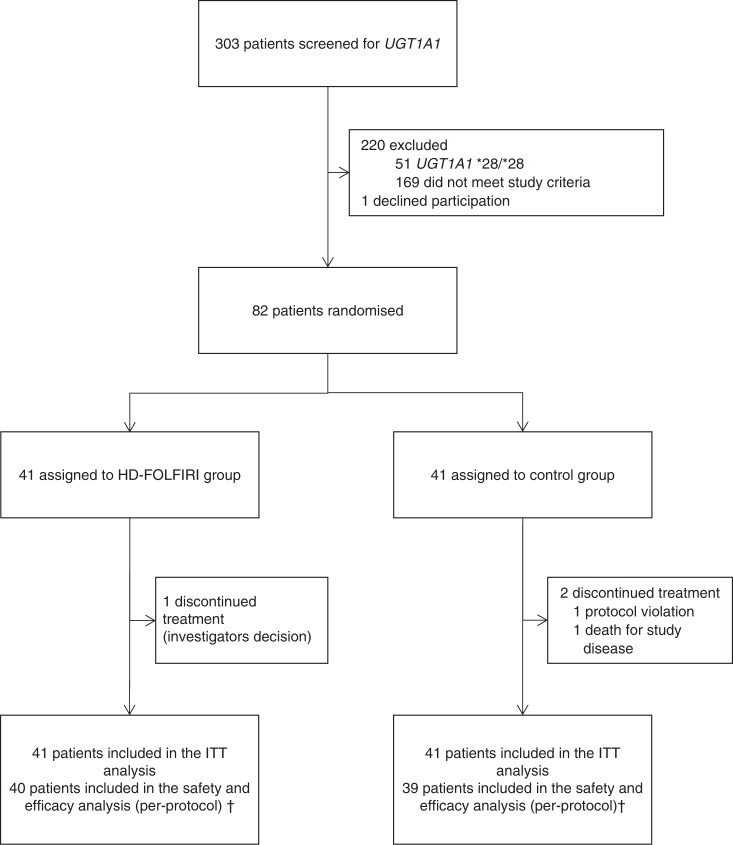
CONSORT diagram

Between June 2012 and October 2016, the *UGT1A1* genotype was analysed in mCRC patients (Fig. 1). Owing to 4 years of slower-than anticipated accrual, the trial was terminated early. Eighty-two patients harbouring UGT1A1*1/*1 or *1/*28 genotypes were enrolled from the three centres, with 41 patients randomly assigned to HD-FOLFIRI (experimental group) and 41 patients to FOLFIRI (control group). Three patients were not included in the final study because they did not receive the pre-planned dose for the endpoint analyses. Therefore, 79 patients were evaluated for toxicity and for response to treatment. Patient characteristics are summarised in Table 1.

**Table 1:** Patient characteristics

	Overall population, *N* = 79^a^	HD-FOLFIRI group, *N* = 40	Control group, *N* = 39	*p* Value
UGT1A1 genotype				
*1/*1	37 (46.8%)	13 (32.5%)	24 (61.5%)	0.01
*1/*28	42 (53.2%)	27 (67.5%)	15 (38.5%)	
Age, years				
Median [range]	63 [40–77]	62 [48–75]	64 [40–77]	0.32
Sex				
Male	49 (62%)	23 (57.5%)	26 (66.7%)	0.41
Female	30 (38%)	17 (42.5%)	13 (33.3%)	
ECOG performance status				
0	38 (48.1%)	17 (42.5%)	21 (53.8%)	0.16
1	39 (49.4%)	23 (57.5%)	16 (41%)	
2	2 (2.5%)		2 (5.1%)	
Primary site				
Right^b^	22 (27.8%)	9 (22.5%)	13 (33.3%)	0.11
Left	36 (45.6%)	16 (40%)	20 (51.3%)	
Rectum	20 (25.3%)	14 (35%)	6 (15.4%)	
Missing	1 (1.3%)	1 (2.5%)	—	
Metastatic development				
Synchronic	62 (78.5%)	31 (77.5%)	31 (79.5%)	0.83
Metachronic	17 (21.5%)	9 (22.5%)	8 (20.5%)	
Number of cycles				
Median [range]	10.6 [3–23]	10.6 [3–23]	10.5 [4–20]	0.9
RAS and BRAF status				
Wild type	36 (45.6%)	18 (45%)	18 (46.1%)	1.00
*RAS* mutant	30 (38%)	15 (37.5%)	15 (38.5%)	
*BRAF* mutant	12 (15.2%)	6 (15%)	6 (15.4%)	
Unknown	1 (1.2%)	1 (2.5%)	—	

^a^Patients who received at least one dose of protocol therapy

^b^Three patients with transverse colon (one in the HD-FOLFIRI group and two in the control group)

### Efficacy

Table 2 shows the results of the tumour response per-protocol analysis. The ORR of patients treated with HD-FOLFIRI was significantly higher than in patients treated with FOLFIRI (67.5% versus 43.6%; *p* = 0.001 odds ratio (OR): 1.73 [95% confidence interval (CI): 1.03–2.93]). In the intention to treat analysis, the ORR was 65.9% in the experimental group versus 43.9% in the control group (*p* = 0.046 OR: 1.64 [95% CI: 0.99–2.72]). There were no interactions between ORR and clinical characteristics (sex, age, ECOG, tumour location, or synchronous disease). Metastatic surgical resection was performed in 15 patients (22.5% in HD-FOLFIRI and 15.4% in FOLFIRI) and was associated with ORR (29.5% versus 5.7%; *p* = 0.007).

**Table 2:** Efficacy data: response rate in the overall population and according to *UGT1A1* and *RAS*/*BRAF* genotypes

	Response rate			*p* Value
	CR + PR (%)	SD (%)	PD (%)	
Overall population, N = 79	44 (55.7)	20 (25.3)	15 (19)	0.001
HD-FOLFIRI group, *N* = 40	27 (67.5)	3 (7.5)	10 (25)	
Control group, *N* = 39	17 (43.6)	17 (43.6)	5 (12.8)	
UGT1A1 genotype				
*1/*1 and *1/*28 N = 79	44 (55.7)	20 (25.3)	15 (19)	0.147
*1/*1 N = 37	17 (46)	13 (35.1)	7 (18.9)	
*1/*28 N = 42	27 (64.3)	7 (16.7)	8 (19)	
RAS and BRAF genotype				
Wild type N = 36	21 (58.3)	9 (25)	6 (16.7)	0.648
*RAS* mutant N = 30	17 (56.7)	6 (20)	7 (23.3)	
*BRAF* mutant N = 12	5 (41.7)	5 (41.7)	2 (16.6)	

There were no interactions between ORR and *UGT1A1* or *RAS* status (Table 2). However, when BRAF mutated tumours were considered, the ORR was 41.7% in the HD-FOLFIRI group versus no objective response in the control group (*p* = 0.003).

### Survival

The median follow-up period was 18 months (range, 1.8–45 months). The cutoff date for PFS and OS was December 2016. At the time of analysis, 89% of the patients had progressed (90% in the experimental group and 87% in the control group). The median PFS and OS were 8.6 and 26 months in the experimental group (HD-FOLFIRI) and 8.2 and 17.6 months in the control group (FOLFIRI) (*p* = 0.46 hazard ratio (HR) 0.84 [95% CI: 0.52–1.35] and *p* = 0.74 HR 0.90 [95% CI: 0.49–1.67], respectively) (Supplementary Figure 1).

PFS was significantly associated with ECOG performance status (9.9 months in ECOG 0 versus 7.2 months in ECOG 1) and metastatic resection (15.5 months in patients who underwent surgery versus 7.8 months in the remaining cases). In terms of OS, patients with metastatic surgery achieved a better outcome than patients who did not undergone surgery (median not reached versus 18.4 months).

No statistically significant difference in PFS or OS was found between the experimental and control groups according to *RAS* or *BRAF* status. In *RAS*/*BRAF* wild-type tumours, the median PFS and OS were 9.8 versus 8.6 months and 29 versus 17 months, respectively. In *RAS*-mutated tumours, the median PFS and OS were 8.5 versus 8.1 months and 14 versus 17.6 months, respectively. In BRAF-mutated tumours, the median PFS and OS were 8.3 versus 7.6 months and 22 versus 11.7 months, respectively.

Table 3 shows the results of the multivariate survival analysis. Multivariate analysis showed a significant association between metastatic resection with both PFS and OS. When patients undergoing metastasectomy were excluded, *BRAF*- and *RAS*-mutated tumours had a shorter PFS and OS than wild-type tumours (Table 4).

**Table 3:** Multivariate survival analysis

	Overall population, *N* = 79	HD-FOLFIRI group, *N* = 40	Control group, *N* = 39	HR [95% CI]	*p* Value
mPFS (months) [95% CI]	8.6 [8–9.2]	8.6 [7.9–9.4]	8.2 [6.8–9.6]	0.84 [0.52–1.35]	0.46
mOS (months) [95% CI]	26 (15–37)	26 [16.7–35.2]	17.6 [1.8–33.4]	0.90 [0.49–1.67]	0.74

**Table 4:** Survival analyses according to RAS and BRAF status

	RAS and BRAF wild type, *N* = 30ª	*RAS* mutant, *N* = 24ª	*BRAF* mutant, *N* = 8ª
mPFS (months) [95% CI]	8.7 [7.2–10.2]	6.7 [3.3–10.2]	4.3 [0.0–10.1]
mOS (months) [95% CI]	25.9 [10.7–41.1]	16.3 [11.3–21.2]	13.8 [1.1–16.5]

^a^Patients undergoing metastasectomy were excluded

### Toxicity

Table 5 summarises the toxicity data available for the 79 patients who received treatment. The non-haematological toxicities that were frequently reported were asthenia and adverse gastrointestinal effects, such as diarrhoea and nausea/vomiting. With regard to haematological toxicities, the most frequent were anaemia, neutropenia, and leukopenia. Most of the toxicities were grade 1 or 2. No significant differences in grade 3–4 toxicities were observed between patients treated with HD-FOLFIRI or standard irinotecan dose (FOLFIRI): diarrhoea (2.5% versus 7.7%), asthenia (5% versus 5.1%), leukopenia (7.5% versus 2.6%), and neutropenia (15% versus 20.5%). Febrile neutropenia was observed in 2 patients in each arm (5% versus 5.1%). No differences were observed in serious adverse events (22.5% versus 20.5%), dose reduction (22.5% versus 28.2%), or prophylactic use of G-CSF (17.5% versus 12.8%) irrespectively of irinotecan dose.

**Table 5:** Safety data

	Overall population		HD-FOLFIRI group		Control group	
	*N* = 79, *N* (%)		*N* = 40, *N* (%)		*N* = 39, *N* (%)	
	All	Grade 3–4	All	Grade 3–4	All	Grade 3–4
Haematological toxicity						
Anaemia	53 (67.1)	1 (1.3)	27 (67.5)	0 (0)	26 (66.7)	1 (2.6)
Leukopenia	21 (26.6)	4 (5.1)	10 (25)	3 (7.5)	11 (28.2)	1 (2.6)
Neutropenia	40 (50.6)	14 (17.7)	20 (50)	6 (15)	20 (51.3)	8 (20.5)
Febrile neutropenia	4 (5.1)	4 (5.1)	2 (5)	2 (5)	2 (5.1)	2 (5.1)
Thrombocythemia	5 (6.4)	0 (0)	0 (0)	0 (0)	5 (12.8)	0 (0)
Non-haematological toxicity						
Diarrhoea	42 (53.2)	4 (5.1)	21 (52.5)	1 (2.5)	21 (53.8)	3 (7.7)
Nausea/vomiting	35 (44.3)	0 (0)	19 (47.5)	0 (0)	16 (41)	0 (0)
Mucositis	25 (31.6)	1 (1.3)	13 (32.5)	1 (2.5)	12 (30.8)	0 (0)
Anorexia	22 (27.8)	0 (0)	8 (20)	0 (0)	14 (35.9)	0 (0)
Asthenia	54 (68.4)	4 (5.1)	26 (65)	2 (5)	28 (71.8)	2 (5.1)

## Discussion

In this study, we continued our line of research by conducting a randomised phase II trial to evaluate the efficacy and safety of the FOLFIRI regimen with HD-FOLFIRI as first-line therapy in patients with mCRC. Our findings confirmed that HD-FOLFIRI increased ORR without adding toxicity in patients with a favourable *UGT1A1* genotype (*1/*1 or *1/*28).

Previous prospective studies have shown that the recommended dose of 180 mg/m^2^ for irinotecan in the FOLFIRI regimen is considerably lower than the dose that can be tolerated by non-UGT1A1*28/*28 mCRC patients. In a Caucasian population study, Toffoli et al.^15^ established that 370 mg/m^2^ in *1/*1 genotype and 310 mg/m^2^ in *1/*28 genotype can be safely administered every 2 weeks in patients undergoing first-line treatment for mCRC treated with FOLFIRI. Similar maximum tolerated doses (MTD) (390 mg/m^2^ in *1/*1 and 340 mg/m^2^ *1/*28 patients) were validated the following year in a study conducted by our group.^14^ In an Asian population, in addition to UGT1A1*28, the variant UGT1A1*6 has been associated with significantly increased related toxicities. In a phase I study, Korean patients were genotyped for *UGT1A1* and stratified according to the number of defective alleles (DA): *28 and/or *6. The recommended doses were 300 (0 DA), 270 (1 DA), and 150 (2 DA) mg/m^2^.^19^

The effect of adding a biologic drug to genotype-guide dosing of FOLFIRI has recently been explored. Two initial retrospective studies in Asian patients were performed by the same group of investigators. They showed that patients with mCRC with pre-therapeutic *UGT1A1* genotyping and subsequent irinotecan dose escalation can achieve a more favourable response and outcome without a significant increase in toxicity while using the FOLFIRI-plus-bevacizumab regimen.^20,21^

More recently, Toffoli et al. published a dose-finding study in first-line mCRC patients treated with FOLFIRI plus bevacizumab to establish the MTD of irinotecan in *1/*1 and *1/*28 patients.^22^ Again, the MTD of irinotecan was 310 mg/m^2^ for UGT1A1*1/*1 patients and 260 mg/m^2^ for *1/*28 patients. The most common dose-limiting toxicities were neutropenia (46%) and diarrhoea (38%), but 65% of the patients treated at the MTD did not require a reduction of irinotecan. These authors also demonstrated that bevacizumab did not alter the pharmacokinetics of irinotecan.

High-dose FOLFIRI (irinotecan 260 mg/m^2^ for UGT1A1*1/*1 and *1/*28 genotypes and 220 mg/m^2^ for UGT1A1*28/*28 genotypes) combined with cetuximab has been explored in a multicentre phase II study (ERBIFORT) in patients with potentially resectable liver metastases of CRC.^23^ This regimen yielded high response rates and enabled complete resection of hepatic metastases in most patients. Thanks to the irinotecan dose adaptation according to *UGT1A1* pharmacogenomics status, this treatment schedule was less toxic yet as effective as the intense combination of FOLFIRINOX plus cetuximab reported by Assenat et al. in a phase II trial.^24^

It is now widely accepted that the dosing recommendations obtained from traditional, non-genotype-directed clinical trials should be revised in light of validated genetic markers of toxicity risk. The lack of patient stratification based on genotype might result in significant underdosing of patient subgroups. However, phase II–III clinical trials are required to determine whether these irinotecan genotype-guided doses—with or without a biological agent—imply a higher antitumour efficacy.

To our knowledge, the present work is the first randomised phase II trial to show higher antitumour efficacy when a genotype-guided dose of irinotecan is considered. It should be noted that this scheme not only increases the response rate and metastasis surgery but also improves efficacy in patients with worse prognosis, such as mutated BRAF. However, the limitations of our study should be considered. In addition to those previously mentioned concerning the early termination due to low accrual, there was an imbalance between experimental and control arms regarding the UGT1A1*1/*1 and *1/*28 genotypes and tumour sidedness, which could have favoured the experimental arm. Therefore, *UGT1A1* genotype and primary tumour location should have been included as stratification factors to avoid bias that could have influenced the results in survival or disease control rates. Moreover, lower doses than in our previous phase I study were used and the FOLFIRI schedule is now given together with biologics in the first-line setting for most mCRC patients, which limits the incorporation of high doses of irinotecan regimens in clinical practice. Regarding toxicity aspects, current evidence highlights the association between dihydropyrimidine dehydrogenase deficiency and an increased risk of fluoropyrimidine-related toxicity.^25^ The additive value of combined *UGT1A1*/*DPYD* genotype analysis could improve the safety of irinotecan plus fluoropyrimidine combinations and should be considered in further studies using these drugs.

To conclude, the findings from our study confirm the safety of chemotherapy with HD-FOLFIRI and indicate that this strategy, although does not show an improvement in survival, improves ORR. A randomised phase II–III study is needed to validate irinotecan intensification according to *UGT1A1* pharmacogenetic status combined with a biological drug adapted to RAS status.

## Electronic supplementary material

Supplementary Figure 1
