# Supplementary material for: Pharmacogenetic clinical randomised phase II trial to evaluate the efficacy and safety of FOLFIRI with high-dose irinotecan (HD-FOLFIRI) in metastatic colorectal cancer patients according to their UGT1A 1 genotype
Source: Br J Cancer. 2018 Dec 26;120(2):190–5. doi: 10.1038/s41416-018-0348-7 (PMC6342907; doi:10.1038/s41416-018-0348-7)
Supplement: Supplementary file 1 — Supplementary Figure 1 [file 41416_2018_348_MOESM1_ESM.docx]

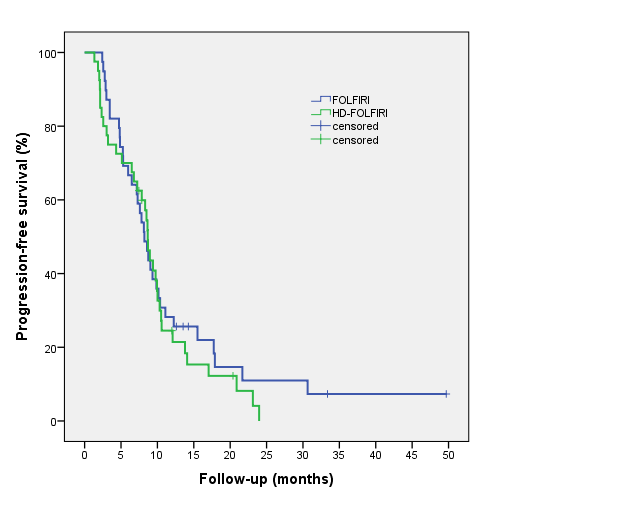

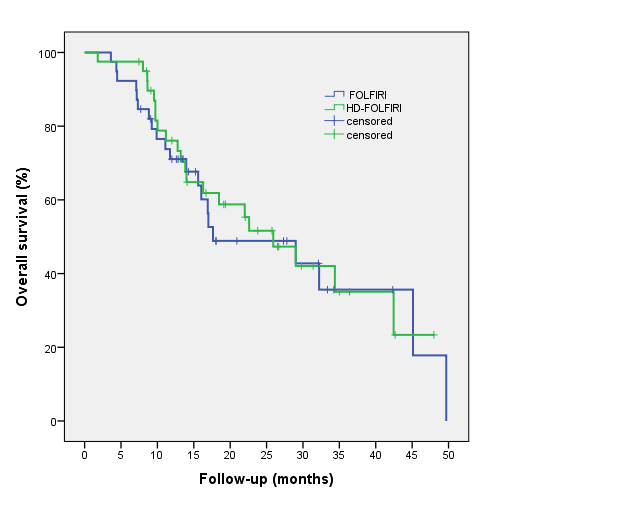


**Number at risk**

**HD-FOLFIRI**

**FOLFIRI**

**Number at risk**

**HD-FOLFIRI**

**FOLFIRI**

40

39

29

29

13

14

5

7

4

4

0

3

0

3

0

1

0

1

0

1

0

0

40

39

39

36

30

28

22

19

17

11

13

10

7

7

5

3

3

3

2

1

0

0

**Hazard ratio 0.84, 95% CI 0.52-1.35**

**p=0.46**

**A**

**B**

**Hazard ratio 0.90, 95% CI 0.49-1.67**

**p=0.74**

Supplementary Figure 1. Kaplan-Meier plots of (A) progression-free survival and (B) overall survival
